# Resident duty hours around the globe: where are we now?

**DOI:** 10.1186/1472-6920-14-S1-S8

**Published:** 2014-12-11

**Authors:** John Temple

**Affiliations:** 1 Research Council, The Healing Foundation, The Royal College of Surgeons, 35–43 Lincoln’s Inn Fields, London, WC2A 3PE, UK

## Abstract

Safe and appropriate health care, especially in urgent or emergency situations, is the expectation of the public throughout the developed world. Achieving this goal requires appropriate levels of medical and other staff, appropriate training, and sensible working hours. Too often the brunt of such care, especially in out-of-hours situations, is borne by medical residents, who – to make matters worse – are frequently poorly supervised by more senior and experienced staff. Many jurisdictions have been alerted to this problem and are striving to correct it. However, the variation in attempts to restrict the actual hours worked by residents to “safe” levels is enormous, and all too often there is no consensus as to what should be put in place to achieve safe patient care. This paper sets out the current position for Europe, North America, and Australia.

## Introduction

The volume of hours worked by medical residents has been a concern for years. The realization that tired, inexperienced, and poorly supervised doctors make more mistakes than those who are fresh, alert, and closely guided has become apparent everywhere. And yet there remains a huge variation in the implementation of controls over the actual hours worked, the environment available for learning, and the degree of real supervision afforded to these young professionals. Variation is seen both between countries with supposedly modern health care delivery systems and within the health systems of those countries themselves.

What should be the role of medical residents? Should they be viewed as *practitioners* primarily, who provide service and attain further learning by clinical exposure (and, some would say, experience), or are they genuinely *doctors in training*, for whom every clinical event should be an appropriately supervised learning opportunity?

The former system has resulted in a random, unstructured, arbitrary, and often patron-dependant method of acquiring the necessary skills to be competent for independent clinical practice. The latter process, which has gained more recognition if not actual implementation in recent times, still has a long way to go before it becomes the accepted and quicker route to senior levels of service and care delivery.

This paper reports on some of the systems and situations around the globe concerning the statutory regulation—or lack of it—as to what constitutes good practice leading to appropriate training of young doctors and, ultimately, safer patient care.

## The statutory position – where there is one

The case of Libby Zion, an 18-year-old woman who died while under the care of residents in a hospital emergency department in New York City in 1984, was the original stimulus to resident duty hour reform [1]. The publicity that surrounded this case highlighted and subsequently influenced attempts to regulate the completely unrestricted hours worked by residents in hospital practice throughout the world [2]. Subsequently, the lead in the journey of restricting hours was taken by Europe. The European Working Time Directive (EWTD), issued by the Council of Europe to protect the health and safety of all workers in the European Union, became law in 1998. It empowered a set of minimum requirements, including the following [3]:

• a maximum work week of 48 hours

• a minimum rest period of 11 consecutive hours per 24-hour duty

• a minimum rest period of 24 hours per 7-day duty, or 48 hours of rest per 14-day duty

• a minimum of 4 weeks of paid annual leave

• a maximum of 8 hours’ work in any 24 hours for workers in stressful positions

• a minimum 20-minute rest period per 6 hours worked

The following section will review the outcome of the EWTD for medical residents since its implementation.

### Europe

Official information remains extremely hard to gather or collate. An official European Union document reporting country-by-country compliance with the EWTD was due for publication in 2008 but has still not been released.

The current situation of the 48-hour EWTD is as follows.

There are beacons of achievement. Denmark has been compliant with the EWTD for many years and has a normal work week of 37 hours. Sweden and Germany indicate good compliance. Finland is probably compliant. The Netherlands reached compliance during 2011. Norway, which is affiliated with the European Union but is not a full member, trains young doctors in a weekly average of 45 hours. The United Kingdom reports compliance now, but recent research [4] suggests that up to 25% of junior doctors are still working beyond the 48-hour limit.

Compliance figures are not available for 11 countries, namely Austria, Belgium, Bulgaria, Cyprus, Czech Republic, Italy, Luxembourg, Malta, Portugal, Slovakia, and Slovenia. There is anecdotal evidence that many doctors in Spain, Ireland, Greece, and France are working more than the regulation 48-hour week, often without additional pay. Poor working conditions and excessive hours, but no hard data, are reported anecdotally in Estonia, Latvia, Lithuania, Poland, and Romania. However, many of this latter group joined the European Union relatively recently and were not previously subject to the EWTD.

In the United Kingdom, the full implementation of the 48-hour EWTD in August 2009 led to widespread concern about the ability of the National Health Service (NHS) to continue to deliver both high-quality training for its staff and safe clinical service. In the health care sector, the EWTD was found to affect only doctors and, more specifically, only those in the secondary care sector. The 2010 report *Time for Training* found that although “high quality training can be delivered in 48 hours” in the NHS, “this is precluded when trainees have a major role in out of hours service, are poorly supervised and access to learning is limited.”[5]

Thus, only 6 of the 27 European member states meet the prescribed standard, some 14 years after the EWTD became a legal requirement. In view of this lack of success, renegotiation of the 48-hour restriction, along with other factors, has been requested, but it will take a very long time for any revision to be agreed, let alone put into practice.

### Canada

In Canada, no national agreement on the reduction of hours has as yet been produced. The Canadian experience with resident duty hours has revolved around the negotiated contracts between residents’ associations, which in this capacity have a function analogous to labour unions, and the provincial jurisdictions in which they train. Currently there is no federal or provincial regulation or statute on the work hours of residents. Nor do the accreditation bodies (Royal College of Physicians and Surgeons of Canada, College of Family Physicians of Canada, and Collège des médecins du Québec) mandate specific limits for duty hours or ensure compliance with the provincially negotiated limits.

The over 10,500 medical residents in Canada are represented by one of seven associations organized under the umbrella of the Canadian Association of Internes and Residents (CAIR) or the Fédération des médecins résidents du Québec (FMRQ). Managed within provincial boundaries, each resident group is a separate negotiating unit. It is this collective bargaining between residents and a multitude of players (e.g., depending on the province, academic health science centres, health boards, ministers of health, medical colleges) that governs duty hours. The contracts are hard to compare directly. Most do not explicitly state a maximum number of hours per week that can be worked. Resident work hours are restricted though the limits placed on consecutive hours of work as well as the frequency of call. The situation is complicated further by differential treatment of in-hospital and out-of-hospital call. Under these contracts it is possible for residents to work up to, and in some cases to exceed, 80 hours per week. Most contracts stipulate a maximum number of consecutive hours that can be worked. This is typically 24 hours, often with some time allowed for handover – typically 2 or more hours (Dr. Salvatore Spadafora, Vice Dean, Postgraduate Medical Education, University of Toronto; personal communication, 2012).

In June 2011, a Quebec labour arbitrator ruled that 24-hour continuous duty assignments pose a danger to residents’ health and therefore violate Section 7 of the Canadian Charter of Rights and Freedoms, which ensures security of the person, and Section 46 of the Quebec Charter of Rights and Freedoms, which requires “fair and reasonable conditions of employment.” The employer concerned was given 6 months to move to a system in which 16-hour shifts are the maximum allowed [6]. This ruling brought considerable public attention to the issue of resident workplace pressures. In December 2011 the FMRQ negotiated an agreement formalizing a maximum of 16 consecutive hours of work for all residents. A pan-Canadian Task Force on Resident Duty Hours has been established by the Royal College of Physicians and Surgeons of Canada to engage the certifying colleges, ministries of health, residents associations, and representatives of academic health sciences centres. This may result in a national consensus.

### The United States

In the United States, the Accreditation Council for Graduate Medical Education (ACGME) certifies resident education as a formal embodiment of professional self-regulation that answers to the profession and the public. In September 2010, the ACGME released new standards [7,8] limiting duty hours for 111,000 residents and subspecialty fellows in 9,000 accredited programs. These requirements, which came into effect in July 2011, include the following:

• a maximum of 80 duty hours per week (averaged over 4 weeks)

• a minimum of 1 day in 7 free of patient care responsibilities (averaged over 4 weeks)

• limitation of in-hospital call to no more than every third night (averaged over 4 weeks)

• a 10-hour period free of duty between duty periods

• a maximum of 4 additional hours for transitioning care

• for first-year post-graduate residents, a maximum shift length of 16 hours, irrespective of the setting (based on research showing increased errors for this group under extended duty periods)

• for intermediate-level residents, a 24-hour limit on continuous duty

The standards also encompass key elements necessary for effective learning and safe patient care, including new standards for supervision, transitions of care, and preparing individuals for learning and practising in teams.

The importance of ensuring the safety of patients in teaching settings has also resulted in the development of enhanced systems for monitoring the new standards, including on-site visits of programs and a new program under which each institution that sponsors residency programs will receive site visits every 18 months.

The outcome of the newly developed ACGME standards is awaited and will be clear only in the years to come.

### Australia

An overview of resident duty hours in Australia is made complex by the number of organizations with legitimate interests in the debate. These include the Commonwealth government and its agencies, state and territorial governments, medico-political bodies such as the Australian Medical Association and various specialty colleges, and entities involved in the design and delivery of education and training and/or the accreditation of education and training programs.

Over the last few years, key messages from a majority of the groups involved are becoming increasingly aligned. Resident and doctor fatigue is a significant safety and quality issue. Many agree that any concerns about reductions in hours worked leading to reductions in competencies gained can be offset by better educational program design and technologies. Australian residents are still working long hours. An Australian Medical Association survey in 2006 reported that 13% of newly qualified doctors and 20% of residents were working “higher risk” hours [9].

In its 2011 report, “A Strategic Study of Postgraduate Medical Training: Baseline Report” [10], Health Workforce Australia foreshadows the rollout of a “scenario tool” that can model what would happen if various factors are changed, including duty hours. This will be of great assistance to all involved in the duty hours discussions and hopefully minimize unintended consequences arising from future reforms. Thus far, however, there is no consensus in Australia about the necessary realignment of resident duty hours [11].

## Discussion

Any requirements for the reduction of resident duty hours presents a challenge to health care systems; any diminution, and especially to 48 hours, requires more doctors to cover the basic 24 hours, particularly in smaller hospitals. It also necessitates a reduction in clinical exposure opportunities. This is especially pertinent to those specialties covering or supporting round-the-clock, 365-day emergency or urgent clinical situations.

A small number of administrations, predominantly in northern Europe, have achieved enviable success in reducing duty hours. Restrictions to 48 hours or less have been confirmed to provide safe care for patients while still achieving satisfactory education and training for staff within an acceptable time frame. These goals seem a long way from fulfilment in the rest of the “developed” world at present.

The fundamental problem is that many, if not most, health care systems rely on physicians who are not yet specialists or consultants to deliver very large amounts of emergency, urgent, or repetitive service, especially in the periods out of regular office hours. Not surprisingly, the impact of duty hour reductions is therefore felt most by those particularly involved in front-door emergency and urgent care and in those disciplines that directly support those services.

Previously, when training was not subject to any time exposure regulation, the opportunity to learn by the experiential model, with (or often without) adequate supervision, usually produced competent and confident doctors. However, the process of acquiring the necessary skills was largely protracted, osmotic, patron-dependant, and quite variable in quality. With appropriate and graded consultant (specialist) supervision, adequate training can be provided in a 48-hour work week. This demands, first, that this consultant workforce be much more hands-on for 24/7 service delivery as and when the service load demands it and, second, that the service environment “makes every moment count” by viewing any clinical exposure for trainees as having the potential to provide a learning experience.

Those disciplines responsible for 24/7 service for emergency and urgent conditions, and the support services assisting this clinical care delivery, are most stressed by the hours reductions, but these are often the very specialties most resistant to change in the mode of service delivery. Such resistance frequently arises either from a fear of more senior doctors being made to be resident when on call or from a conviction that “what was good for us is good for those following behind.” The number of consultants in the United Kingdom increased by more than 60% from 2000 to 2010. The number of more junior doctors employed was also increased in the same proportion by stepping up the output and number of medical schools and through very active recruiting of foreign medical graduates. The result has been that methods of training and service delivery did not change. Hence the main conclusion of *Time for Training*, highlighted earlier.

The resident-on-call role for senior doctors is surely necessary only where the service load dictates that it is sensible and safe for patient care (usually in very high service load situations). Adequate planning in such situations will help to foster a better work–life balance for all levels of staff.

To achieve significant reductions in duty hours for doctors in training, structural changes must be made to the way service and concomitant training are delivered. This is a message that can be applied universally. Consultants must be more directly responsible for the delivery of all types of care, at all times. To make this possible, enough trained doctors need to be available in any given service setting. The clinical team then needs to be of sufficient size, at senior and junior levels, to allow rotas that are compliant and lead to a healthy work–life balance. Remembering that these senior doctors themselves are subject to the same hours restrictions, there must be a large enough throughput of clinical material to provide a satisfactory, engaging workload and maintain the necessary skills for safe practice. Where this is not possible, the scene is set for the reorganization and amalgamation of services and hospitals, and this must include reassigning clinical services to other trained professional groups, such as nurse practitioners and physicians’ assistants, within a teamwork setting.

Although Europe aims to maintain a maximum 48-hour work week for all health care workers, in North America the standard the authorities state they are trying to reduce to is 80 hours per week – the so called “safe hours.” Yet, because of the lack of any enforceable consensus, it is still not uncommon in many places for residents to work in excess of 100 hours per week.

The basic reason why agreement to significant change has been possible in some European countries is that the negotiations have been carried out by single organizations on behalf of the medical staff. In the United Kingdom, for instance, the British Medical Association provides a strong, unified voice to government. Where multiple bodies are involved, as in any federal system, then final agreement is much more difficult to achieve. The United States, Canada, and Australia are good examples of this type of process resulting in failure of agreement and progress to date.

## Conclusion

The defined goal must be a reduction in working hours for all doctors at all levels in the hospital services to a level sustainable for a healthy work–life balance for staff and safe care for patients. Tired, inexperienced, and poorly supervised junior doctors make more mistakes than both those who are more rested and adequately supervised, or more senior, and therefore knowledgeable and experienced, practitioners.

What is apparent at present is that there is no uniform formula or practice anywhere in the so-called “western” world. Europe has attempted to set statutory thresholds but has succeeded in applying these in fewer than 25% of member states so far. Only the Scandinavian countries have achieved systems of significantly reduced hours, often to fewer than 40 per week, without detriment to service delivery or to the education and training of young doctors. The United Kingdom and several other European countries are close to achieving compliance with the 48-hour week, and thus more sensible resident duty hours, but most of the rest of Europe has a long way to go. North America and Australia are currently far in excess of this level and currently consider an 80-hours work week “safe.” Even in Germany during the height of the bombing campaign by the RAF and the USAF in 1943 and 1944, which was aiming to paralyze the infrastructure of the Third Reich, the work week for aircraft production workers was increased to only 72 hours! [12]

In most health care systems, the correct adjustment of duty hours will be achieved only by a combination of sensible, agreed working practices coupled with service reorganization, which will inevitably mean fewer larger hospitals and enhanced community care and patient transport services. There is a long way to go in most countries before this goal can be reached.

## Competing interests

The author declares he has no competing interests.

## Declarations

Resources and secretariat support for this project was provided by the Royal College.

This article has been published as part of *BMC Medical Education* Volume 14 Supplement 1, 2014: Resident duty hours across borders: an international perspective. The full contents of the supplement are available online at http://www.biomedcentral.com/bmcmededuc/supplements/14/S1. This article was submitted in 2011 and peer reviewed in 2012. Final acceptance for publication as part of this supplement was in 2014.

Publication of this supplement was supported and funded by the Royal College of Physicians and Surgeons of Canada. The funding agency played no role in the design, in the collection, analysis and interpretation of data; in the writing of the manuscripts; and in the decision to submit the manuscript for publication. The articles have been through the journal's standard peer review process for supplements. The Supplement Editors declare that they have no competing interests.
